# Postprandial Dynamics of Proglucagon Cleavage Products and Their Relation to Metabolic Health

**DOI:** 10.3389/fendo.2022.892677

**Published:** 2022-06-29

**Authors:** Robert Wagner, Sabine S. Eckstein, Louise Fritsche, Katsiaryna Prystupa, Sebastian Hörber, Hans-Ulrich Häring, Andreas L. Birkenfeld, Andreas Peter, Andreas Fritsche, Martin Heni

**Affiliations:** ^1^ Institute for Diabetes Research and Metabolic Diseases of the Helmholtz Center Munich at the University of Tübingen, Tübingen, Germany; ^2^ German Center for Diabetes Research (DZD), Tübingen, Germany; ^3^ Department of Internal Medicine IV, Division of Diabetology, Endocrinology, and Nephrology, Eberhard Karls University Tübingen, Tübingen, Germany; ^4^ Department of Endocrinology and Diabetology, Medical Faculty and University Hospital, Heinrich Heine University, Düsseldorf, Germany; ^5^ Institute for Clinical Diabetology, German Diabetes Center (DDZ), Leibniz Center for Diabetes Research at Heinrich-Heine University, Düsseldorf, Germany; ^6^ Institute for Clinical Chemistry and Pathobiochemistry, Department for Diagnostic Laboratory Medicine, University Hospital Tübingen, Tübingen, Germany; ^7^ Division of Endocrinology and Diabetology, Department of Internal Medicine 1, University Hospital Ulm, Ulm, Germany

**Keywords:** glucagon, Glucagen-like peptides, insulin, metabolism, glicentin

## Abstract

**Introduction:**

While oral glucose ingestion typically leads to a decrease in circulating glucagon levels, a substantial number of persons display stable or rising glucagon concentrations when assessed by radioimmunoassay (RIA). However, these assays show cross-reactivity to other proglucagon cleavage products. Recently, more specific assays became available, therefore we systematically assessed glucagon and other proglucagon cleavage products and their relation to metabolic health.

**Research Design and Methods:**

We used samples from 52 oral glucose tolerance tests (OGTT) that were randomly selected from persons with different categories of glucose tolerance in an extensively phenotyped study cohort.

**Results:**

Glucagon concentrations quantified with RIA were non-suppressed at 2 hours of the OGTT in 36% of the samples. *Non-suppressor*s showed lower fasting glucagon levels compared to *suppressors* (p=0.011). Similar to RIA measurements, ELISA-derived fasting glucagon was lower in *non-suppressors* (p<0.001). Glucagon 1-61 as well as glicentin and GLP-1 kinetics were significantly different between *suppressors* and *non-suppressors* (p=0.004, p=0.002, p=0.008 respectively) with higher concentrations of all three hormones in *non-suppressors*. Levels of insulin, C-peptide, and free fatty acids were comparable between groups. *Non-suppressors* were leaner and had lower plasma glucose concentrations (p=0.03 and p=0.047, respectively). Despite comparable liver fat content and insulin sensitivity (p≥0.3), they had lower 2-hour post-challenge glucose (p=0.01).

**Conclusions:**

Glucagon 1-61, glicentin and GLP-1 partially account for RIA-derived glucagon measurements due to cross-reactivity of the assay. However, this contribution is small, since the investigated proglucagon cleavage products contribute less than 10% to the variation in RIA measured glucagon. Altered glucagon levels and higher post-challenge incretins are associated with a healthier metabolic phenotype.

## Introduction

**Figure 1:**
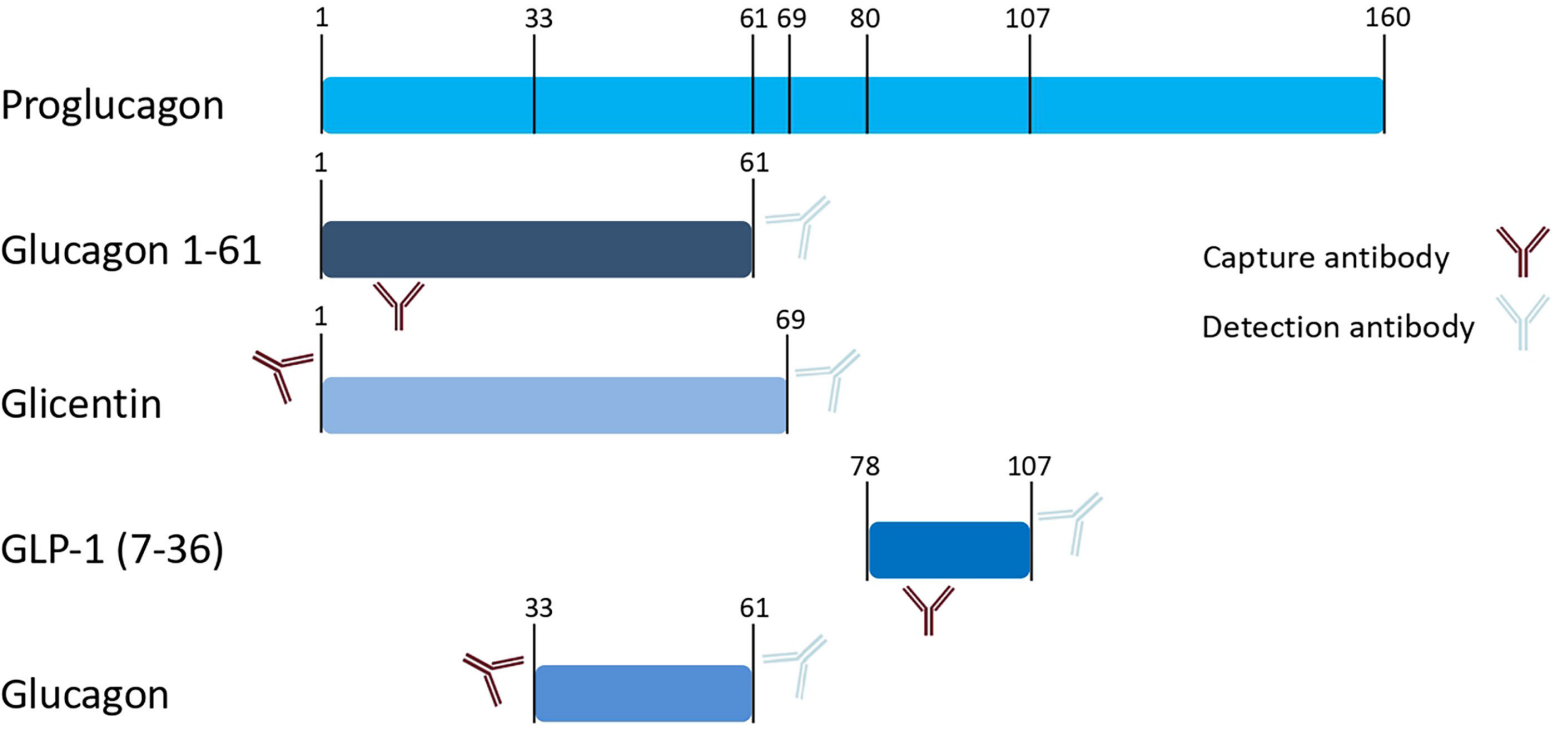
Schematic presentation of proglucagon and proglucagon cleavage products. Numbers indicate amino acid positions of cleavage sites. Antibodies schematically indicate epitopes that are used by the commercial immunoassays applied in our measurements (as provided by the manufacturer).

Glucagon, glicentin, and glucagon-like peptide (GLP)-1 as proglucagon cleavage products all originate from the same preproglucagon gene (see **Figure 1**). Differential expression of the preproglucagon gene in varying tissues is accompanied by differential processing of the proglucagon transcript by prohormone convertases.

For a long time, counteracting insulin was thought to be the main function of glucagon. Since patients with type 2 diabetes show elevated fasting glucagon levels (1), the hormone has been believed to be a relevant contributor to hyperglycemia. Research over the last years, however, revealed a much more complex role of glucagon with potentially beneficial effects for whole-body metabolism (2, 3).

GLP-1 is an insulinotropic peptide that potentiates insulin secretion and mainly originates from the L cells in the distal small bowel and colon (4, 5). Glicentin is also produced in the intestinal L cells and its exact functions are still enigmatic. Studies in animal models and *in vitro* human tissues suggest a regulatory function on intestine, gastric acid production and insulin production (6–12).

Studies have shown that bariatric surgery for the treatment of obesity results in dramatic metabolic changes which can at least partially be attributed to incretins such as GLP-1 and glicentin (13–15). Uncovering the complex regulation that underlies the beneficial effects of bariatric surgery requires a better understanding of different incretins and their interplay on metabolism.

In a multi-cohort study with more than 4000 participants who underwent oral glucose tolerance tests, we found non-suppressed glucagon levels at 2-hours after glucose load in 21-34% of the study populations (16). Surprisingly, this non-suppression of glucagon was associated with a metabolically healthier phenotype. One limitation of the findings was the radioimmunoassay used to quantify glucagon in the study as this assay is known for substantial cross-reactivity with other proglucagon cleavage products (17), at least in an updated version marketed since around 1999 (18). This is caused by the overlapping amino acid sequences of glucagon with other incretin peptides, resulting in assay cross-reactivity when the capture antibody binds to an epitope within these shared sequences. The proglucagon sequence from amino acid position 33-61 corresponds to glucagon. The alternative gene product glicentin spans amino acids 1-69 of the proglucagon gene, whereas GLP-1 comprises amino acids 78-107 (**Figure 1**). Wewer-Albrechtsen et al. investigated glucagon 1-61, which is another circulating proglucagon fragment (spanning the proglucagon sequence 1-61). Glucagon 1-61 has been shown to stimulate insulin secretion and act on human hepatocytes in a series of experiments (19). Glucagon 1 – 61 comprises the amino acid sequence of glucagon and glicentin but lacks the eight C-terminal amino acids of glicentin (19) (**Figure 1**).

Due to a cross-reactivity of the radioimmunoassay, it is plausible that the presumed positive effects of glucagon on metabolism we observed in our previous study result from effects of other proglucagon fragments. Therefore, we aimed to investigate the post-load dynamics of glucagon and it’s with other proglucagon fragments quantified with a highly specific glucagon immunoassay.

## Results

**Figure 2:**
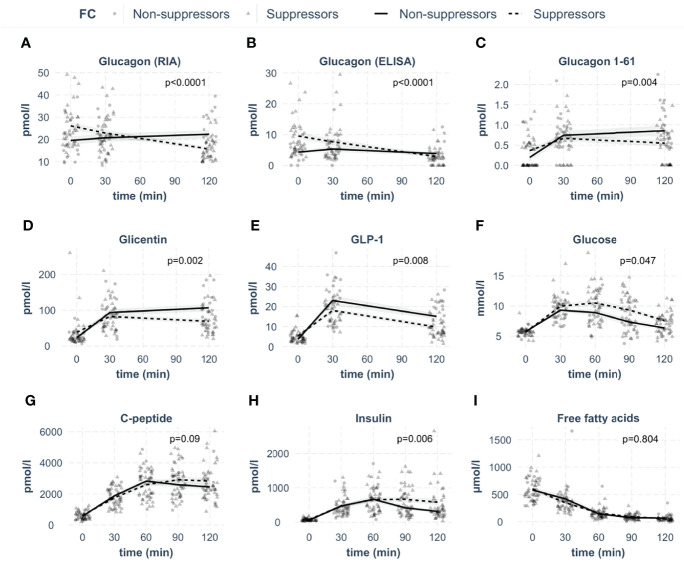
Concentrations of investigated analytes in the groups of glucagon *suppressors* and *non-suppressors* during the OGTT. The respective analyte is indicated in the box. (**A**: glucagon measured by radioimmunoassay, **B**: glucagon measured by ELISA, **C**: glucagon 1-61, **D**: glicentin, **E**: GLP-1, **F**: glucose, **G**: C-peptide, **H**: insulin, **I**: free fatty acids). Lines represent means with standard errors. Circles indicate data points for suppressors, triangles for non-suppressors, p-values were calculated with linear mixed models. N=52.

**Figure 3:**
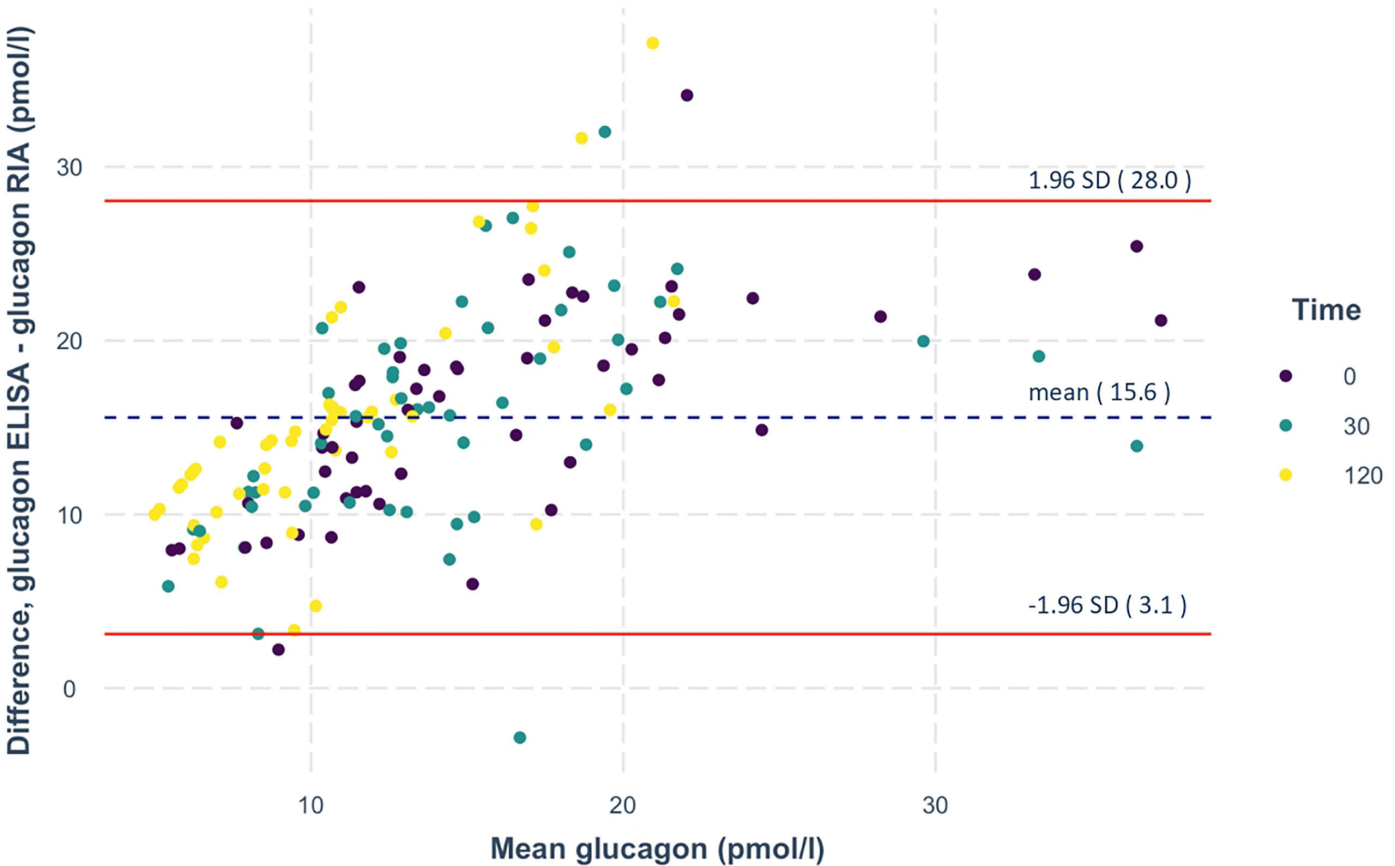
Bland-Altman plot of the RIA- and ELISA-measured glucagon. Differences in glucagon measurements between the two assays are plotted against mean glucagon values. The dashed lines represent the mean, the solid lines depict the lines of agreement calculated as mean ± 1.96 times the SD of this difference. N=156 measurements from 52 oGTTs.

Glucagon concentrations were first measured by radioimmunoassay (**Figure 2A**). Analyzing these measurements, 36% of the participants showed stable or rising RIA-derived glucagon concentrations during the OGTT. We defined them as *non-suppressors*. Of note, the *non-suppressors* had significantly lower fasting levels of glucagon compared to the *suppressors* (p=0.011). In parallel, samples were measured with the highly specific glucagon ELISA, showing lower values compared to RIA. Bland-Altman plots revealed reasonable agreement between the two methods with on average 15 ± 6 pmol/l higher concentrations from RIA measurements (**Figure 3**).

The ELISA also detected persons with stable or rising glucagon. Similar to RIA measurements, fasting glucagon was lower in the *non-suppressors* (p<0.001). The RIA- and ELISA-derived glucagon *suppressors* and *non-suppressors* showed similar kinetics, regardless of which assay the hormone was measured with (**Figures 2A, B**). As the applied glucagon RIA is known to have cross-reactivity with other proglucagon cleavage products (18, 20, 21), we quantified these hormones by highly specific ELISAs. We measured glucagon 1-61, glicentin and GLP-1 from the same samples (**Figures 2C–E**).

**Figure 4:**
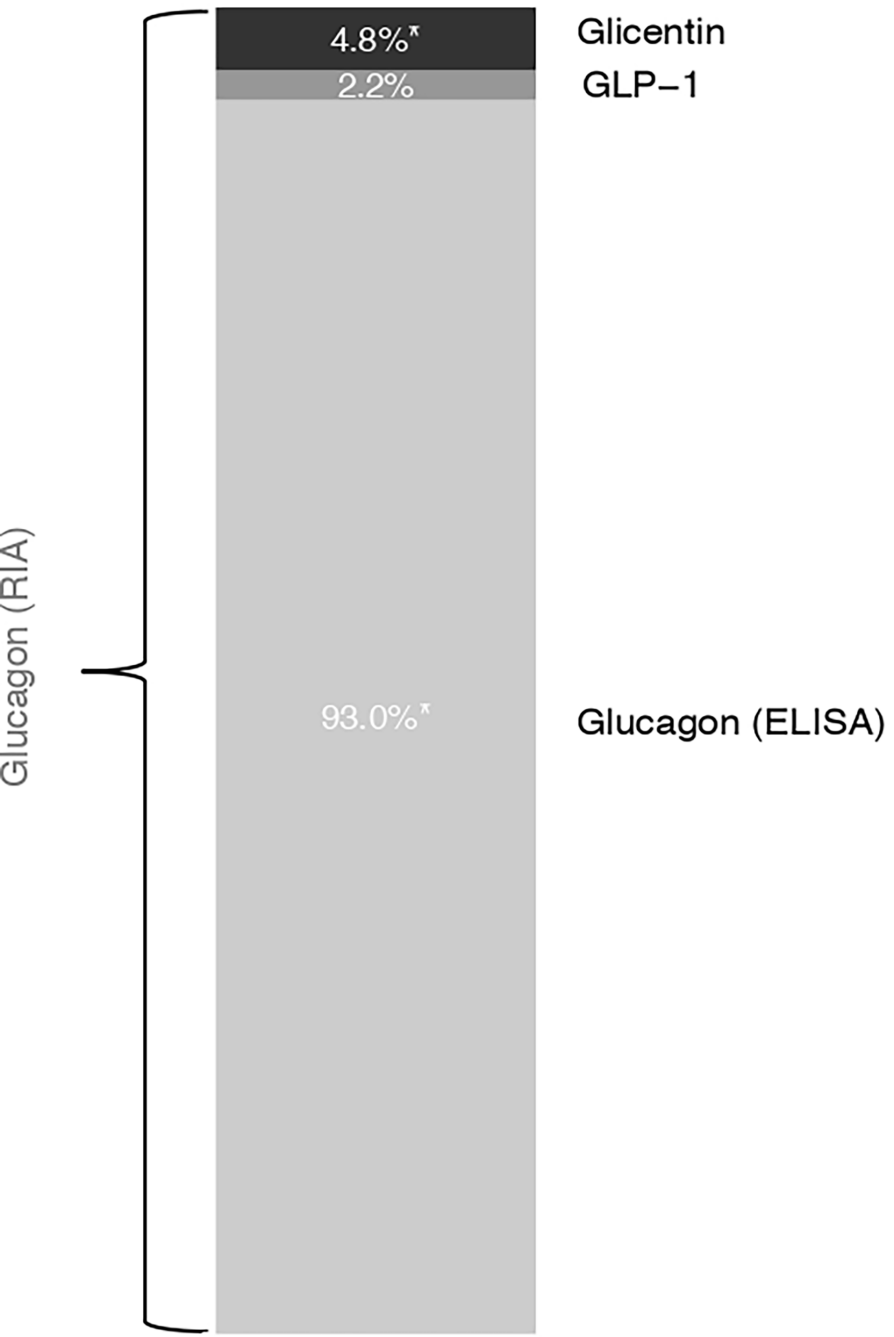
Estimated model-based relative contribution of proglucagon cleavage products to the variance of RIA-derived glucagon measurements. We used linear mixed models with the participant as a random intercept and the OGTT-time point as fixed effects. Marginal R squared was calculated to describe the proportion of variance in the outcome variable explained by the fixed effect. Removing each factor separately, we determined the percentage of its respective contribution to RIA-derived glucagon measurements that are presented here as a bar chart.

To investigate the relative contribution of these proglucagon cleavage products to RIA-derived glucagon measurements, we modeled glucagon RIA levels at all available OGTT time points using glucagon ELISA, glicentin, glucagon 1-61 and GLP-1. The share of total variance explained by this model was 82.2%. The combination of the above-mentioned variables explained 43.6% as fixed effects. By removing each factor separately, we determined their relative contributions. In **Figure 4**, we show the relative contribution of glicentin, GLP-1 and ELISA-based glucagon to the total variance of the RIA-based glucagon measurement (total variance was set to 100%). ELISA measured glucagon explained 93% of the variance, whereas glicentin explained 5% of it. The contribution of GLP-1 (2%) was not statistically significant. Inclusion of Glucagon1-61 in the model deteriorated its performance and has been therefore removed from the final model.

While the fasting concentration of glucagon 1-61 and glicentin were comparable between groups (p≥0.1), GLP-1 was significantly lower in *non-suppressors* (p=0.02).

Glucagon 1-61 as well as glicentin kinetics were significantly different between *suppressors* and *non-suppressors* with higher concentrations of both hormones in *non-suppressors* (**Figures 2C, D**). For GLP-1, the concentrations were not significantly different (**Figure 2E**).

As incretins stimulate pancreatic insulin release, we next analyzed insulin and C-peptide levels as well as plasma glucose. Insulin and C-peptide levels were comparable between the two groups (**Figures 2G, H**). However, insulin concentrations were lower in the *non-suppressors* in the last 30 minutes of the OGTT, resulting in a statistical difference between groups. In line with comparable insulin secretion, suppression of free fatty acids was not different between groups (**Figure 2I**). However, plasma glucose concentrations were significantly lower in the *non-suppressors* (**Figure 2F**), even after adjustment for BMI (p=0.04).

The *non-suppressors* were leaner (p=0.03) and had a lower waist circumference (p=0.04). Despite comparable liver fat content and insulin sensitivity (p≥0.3), they had lower post-challenge glucose concentrations (p=0.01), which remained significant after adjustment for BMI (p=0.002).

## Discussion

In a meta-analysis with over 4000 participants, we previously identified non-suppression of glucagon in response to oral glucose intake, to be linked to a favorable whole-body metabolism (16). One limitation of that study was quantification of glucagon by RIA that likely cross-reacts with additional proglucagon cleavage products. We therefore performed comparative analyses of glucagon kinetics assessed by RIA and a highly sensitive ELISA. In addition, we investigated the kinetics of the major proglucagon cleavage products glucagon 1-61, glicentin and GLP-1. Regardless of the measurement approach, we identified persons with glucagon suppression during the OGTT and such with stable or even rising concentrations. The persons that did not suppress RIA-measured glucagon in response to oral glucose intake showed higher incretin responses during the glucose challenge. Of note, these differences in glucagon and incretin kinetics were accompanied by a metabolically healthier phenotype.

Our data suggest that glucagon 1-61, glicentin and GLP-1 could partially account for RIA-derived glucagon measurements. However, this contribution appears to be small, because *(i)* there is a reasonable agreement between RIA and ELISA measurements and *(ii)* the investigated proglucagon cleavage products explain less than 10% of total glucagon measured with RIA.

There is substantial controversy on the role of glucagon in the pathogenesis of diabetes (22, 23). While glucagon promotes hepatic glucose production, this effect is self-limited, and compensated by glucagon’s stimulation of insulin secretion, inhibition of hepatic triglyceride synthesis, increase in basal energy expenditure and central inhibitory effects on appetite (3, 23, 24). Glucagon also stimulates GLP-1 receptors, though, to a lesser extent than its primary ligand (25), that has been proven beneficial in the treatment of both diabetes and obesity. Thus, although there is a clear association of fasting hyperglucagonemia and type 2 diabetes (26, 27), this might be secondary to compensatory processes without playing a causal role in diabetes development. When it comes to postprandial changes in glucagon concentrations, results are even more puzzling (16, 28–30) and the detailed contribution in the pathogenesis of type 2 diabetes is still unclear (22). While glucagon non-suppression in the postprandial state was long thought to be a hallmark of impaired glucose tolerance, based on smaller studies (31, 32), this did not hold true in our large meta-analysis (16), where post-load non-suppression of glucagon was detected in those with a healthier phenotype.

In our current study, we replicated this finding with the highly specific glucagon immunoassay and extended these findings by quantifying additional proglucagon cleavage products. Higher post-load levels of glucagon 1-61, glicentin and GLP-1 in the glucagon non-suppressor group were associated with lower blood glucose levels. Given the insulinotropic effect of incretins, the most obvious explanation could be a difference in insulin secretion between the two groups. However, our data argue against this, as we detected no significant difference in C-peptide levels, which are indicative for pancreatic insulin secretion. This is further underlined by comparable insulin-induced suppression of lipolysis, which was assessed by free fatty acid concentrations.

Fasting glucose, that strongly relies on endogenous glucose production (33) is not different between the *suppressor* and *non-suppressor* glucagon groups. This suggests that endogenous glucose production does not have a relevant contribution to differences in glycaemia between the two groups. Comparable whole-body insulin sensitivity argues against major differences in hepatic insulin sensitivity that would result in different suppression of endogenous glucose production during the OGTT.

One possible explanation for the altered glucose levels is therefore a difference in non-insulin dependent glucose disposal. Non-insulin dependent glucose disposal is also referred to as glucose sensitivity or glucose effectiveness. This term describes the ability of glucose to regulate glucose disposal and gluconeogenesis by itself under basal insulin conditions (34). Tissues such as fat and muscle usually take up glucose in an insulin-dependent fashion. However, insulin-independent glucose uptake is also present (35, 36). Glucagon has direct glucoregulatory effects through the brain (24). Thus, one possibility is that glucose effectiveness is regulated *via* the brain, though the molecular mechanisms are still unclear (37). Our results suggest that glucagon or incretins could be involved and may promote non-insulin dependent glucose disposal. This mechanism could contribute to the glucose-lowering effect of these hormones and to recent pharmacotherapies that target these pathways.

Apart from its classical hyperglycemic potency, glucagon has pleiotropic effects on appetite, energy metabolism and hepatic triglyceride synthesis. To exploit the beneficial effects while compensating for hyperglycemic effects, GLP-1 and glucagon coagonists are already being clinically tested for the treatment of diabetes and non-alcoholic steatohepatitis. Since levels increase postprandially, one mechanistic explanation for the association of increased post-challenge glucagon with metabolic health could be a physiologic co-agonism of GLP-1 and glucagon.

Our study is limited by the sample size and the fact that we did not apply mass spectrometry as gold standard for the identification of peptide hormones. We also did not measure all proglucagon cleavage products that could cross-react with the glucagon radioimmunoassay, including oxyntomodulin (18). In addition, we did not test additional stimuli that potentially trigger gastrointestinal secretion of proglucagon cleavage products, e.g. mixed meal test.

In summary, we verified that in some persons oral glucose intake does not result in a suppression of glucagon. Of note, these persons additionally displayed higher post-load concentrations of further preproglucagon cleavage-products, including glicentin. As these persons are leaner and have lower blood glucose, our results indicate that proglucagon cleavage-products could potentially contribute to the development of a healthier metabolic phenotype.

## Methods

### Subject Characteristics and Measurements

Samples from 52 randomly selected participants of the prediabetes lifestyle intervention study (38) (PLIS, registered with clinical trial.gov NCT01947595) were investigated in this project. None of the study participants took any kind of medication that interferes with glucose or energy metabolism. The study was approved by the local ethics board (Ethics Committee of the Medical Faculty of the Eberhard Karls University and the University Hospital Tübingen) and conducted in accordance with the declaration of Helsinki. All participants provided written informed consent.

Oral glucose tolerance tests (OGTT) were performed after an overnight fasting period of 12 hours. Venous blood was drawn at baseline and 30, 60, 90 and 120 minutes after drinking a 75 g glucose solution (Accu-Check Dextrose O.G.T., Roche Diagnostics, Mannheim, Germany). Plasma glucose and free fatty acids were measured from sodium fluoride plasma with an ADVIA chemistry XPT autoanalyzer (Siemens Healthineers). Serum insulin and C-peptide were analyzed using ADVIA Centaur XPT immunoassay system (Siemens Healthineers). Study participants were categorized into glycemic groups: normal fasting glucose, impaired fasting glucose, impaired glucose tolerance, impaired fasting glucose + impaired glucose tolerance, and type 2 diabetes according to ADA criteria (39). For details see **Table 1**.

**Table 1:** Subject characteristics.

	Stratified by glucagon RIA dynamics		P _unadjusted_	^1^P _adjusted_
	Suppressors	Non-suppressors		
n	33	19		
Age (years)	59 (9)	61 (11)	0.397	
BMI (kg/m^2^)	29.3 (5.1)	26.5 (3.4)	0.034*	
Sex (f/m)	14/19	9/10	0.956	
^2^NFG/IFG/IGT/IFG+IGT/DIA	9/10/3/7/4	6/10/0/2/1	0.365	
Insulin sensitivity index, OGTT-derived (AU)	10.9 (9.7)	12.0 (5.6)	0.633	0.572
^3^Liver fat content (%)	6.8 (8.0)	4.9 (4.7)	0.389	0.544
HbA1c (%)	5.8 (0.5)	5.7 (0.3)	0.499	0.832
Fasting glucose (mmol/l)	5.7 (0.63)	5.8 (0.76)	0.635	0.376
2-hour glucose (mmol/l)	7.6 (1.8)	6.4 (1.2)	0.010*	0.002*
Waist circumference (cm)	100.0 (15.9)	91.3 (9.7)	0.035*	0.566
Hip circumference (cm)	105.9 (9.8)	101.3 (7.2)	0.077	0.981
Waist-to-Hip-Ratio (WHR)	0.94 (0.1)	0.90 (0.1)	0.129	0.613

Numbers in columns represent mean and standard deviation. * denotes statistical significance.

^1^adjusted for BMI and sex.

^2^Normal fasting glucose (NFG), impaired fasting glucose (IFG), impaired glucose tolerance (IGT), participants with T2D (DIA).

^3^ Total measurements are 44, for “Suppressors” n=28, for “Non-suppressor” n=16.

Glucagon was measured by a commercially available radioimmunoassay (Linco Research/Millipore, St. Charles, MO). Glucagon, glicentin and GLP-1 were also measured with commercially available ELISA assays (Mercodia, Uppsala, Sweden) according to the manufacturer’s instructions. We did not use the novel extended washing protocol that had been recommended for glucagon measurements in patients after bariatric surgery. The antibody binding sites (19, 40) are indicated in **Figure 1**. Glucagon 1-61 was measured with a prototype ELISA from Mercodia (Uppsala, Sweden) according to the manufacturer’s instructions. The prototype ELISA shows 0.5% cross-reactivity with glicentin and < 0.2% with glucagon respectively. To ensure optimal sample quality, EDTA plasma was stabilized with 300 ng/ml protease inhibitor aprotinin (Sigma, Merck, Darmstadt, Germany) und subsequently processed at 4°C and kept frozen at -80°C until batch measurement.

HbA1c was measured by HPLC. Height, weight, waist and hip circumference were measured according to standard operating procedures. Liver fat content was measured by localized (1)H-MR spectroscopy using a 1.5 T MR scanner (Magnetom Sonata, Siemens Healthcare, Erlangen, Germany) and the distribution of lean and adipose tissues was measured by whole body MR imaging as previously described (41).

### Calculations

Insulin sensitivity was assessed with the index proposed by Matsuda and De Fronzo (42).

### Statistics

The relative change of glucagon RIA from 0 to 120 min was used to define the groups of *suppressors* and *non-suppressors*. The *suppressors* were determined as participants with the fold change glucagon RIA (glucagon RIA 120 min/glucagon RIA 0 min) less than 1, while *non-suppressors* were those with equal or higher than 1.

To analyze measurements from the same participants at different time-points of the OGTT, linear mixed models with the participant as random intercept and the OGTT-time point as fixed effects were used. By constructing a model with RIA glucagon as outcome and glicentin, ELISA-glucagon, GLP-1 as explanatory variables, also accommodating the time-point of measurement (i.e. 0, 30 or 120 minutes) in a linear mixed model, we estimated the contribution of these variables to the measured RIA glucagon *in vivo*, during OGTT. To this end, the marginal coefficient of determination (pseudo R-squared) was calculated for mixed models to describe the proportion of variance in the outcome variable explained by the fixed effect using the MuMIn package in R. Removing each factor separately, we estimated the percentage of its respective contribution to RIA-derived glucagon measurements.

The Wilcoxon rank-sum/Kruskal-Wallis test was used to perform nonparametric tests on continuous variables, for comparing two independent samples. Categorical variables were compared by chi-squared test. P-values < 0.05 were considered statistically significant. Statistical analyses were performed with R (version 4.0. 3) (43).

## Data Availability Statement

The data is not publicly available due to containing information that could compromise research participant privacy/consent.

## Ethics Statement

The studies involving human participants were reviewed and approved by Ethics Committee of the Medical Faculty of the Eberhard Karls University and the University Hospital Tübingen. The patients/participants provided their written informed consent to participate in this study.

## Author Contributions

RW, SE, LF and MH designed the analysis strategy, supervised the project and contributed to discussion. SE, LF, SH and AP performed measurements and contributed to discussion. LF and KP did statistical analysis. H-UH, AB and AF contributed to discussion. RW, SE and MH drafted the manuscript. All authors approved the final version of the manuscript prior to submission.

## Funding

We acknowledge support by the Deutsche Forschungsgemeinschaft and the Open Access Publishing Fund of the University of Tübingen. This work was supported in part by grant 01GI0925 from the German Federal Ministry of Education and Research (BMBF) to the German Center for Diabetes Research (DZD).

## Conflict of Interest

Outside of the current work, RW does report lecture fees from Novo Nordisk, Sanofi-Aventis and travel grants from Eli Lilly. He served on the advisory board for Akcea Therapeutics, Daiichi Sankyo, Sanofi-Aventis and NovoNordisk. Outside of the current work, AF reports lecture fees and advisory board membership from Sanofi, Novo Nordisk, Eli Lilly, and AstraZeneca. In addition to his current work, AB reports lecture fees from AstraZeneca, Boehringer Ingelheim, and NovoNordisk. He served on advisory boards of AstraZeneca, Boehringer Ingelheim and NovoNordisk. Outside of the current work, MH, reports research grants from Boehringer Ingelheim and Sanofi (both to the University Hospital of Tübingen), advisory board for Boehringer Ingelheim, and lecture fees from Amryt, Novo Nordisk and Boehringer Ingelheim.

The remaining authors declare that the research was conducted in the absence of any commercial or financial relationships that could be construed as a potential conflict of interest.

## Publisher’s Note

All claims expressed in this article are solely those of the authors and do not necessarily represent those of their affiliated organizations, or those of the publisher, the editors and the reviewers. Any product that may be evaluated in this article, or claim that may be made by its manufacturer, is not guaranteed or endorsed by the publisher.
